# Early dismantling of the polyethylene liner in the Arpe® trapezial cup: a report of two cases

**DOI:** 10.1177/17531934211012203

**Published:** 2021-04-29

**Authors:** Rasmus D. Thorkildsen, Ole Reigstad, Magne Røkkum

**Affiliations:** 1 Division of Orthopaedic Surgery, Oslo University Hospital, Oslo, Norway; 2 Institute of Clinical Medicine, University of Oslo, Oslo, Norway

Dear Editor,

The Arpe® prosthesis (Zimmer Biomet, Warsaw, IN, USA) for trapeziometacarpal replacement is an unconstrained ball-and-socket implant with a thin non-exchangeable polyethylene (PE) liner inserted into the trapezial cup and a stemmed metacarpal component. The thin liner is secured with a peg through a hole in the cup’s metal backing. We report an unusual complication in two mechanics treated with an Arpe® prosthesis for trapeziometacarpal osteoarthritis.

A 49-year-old man complained about increasing activity-related pain at the base of his dominant thumb 3.5 years after implantation of an Arpe® prosthesis. There was no local swelling, redness or increased temperature. Grinding and palpation elicited pain. Radiographs were normal. CT scans revealed the head of the implant slightly off centre. At revision, we found the liner loose from the well-anchored metal backing (Figure 1). The cup size 9 was exchanged to the larger size 10. After 6 weeks of cast immobilization, he gradually resumed daily activities over 6 weeks and work activities by 3 months postoperatively.

**Figure 1.**
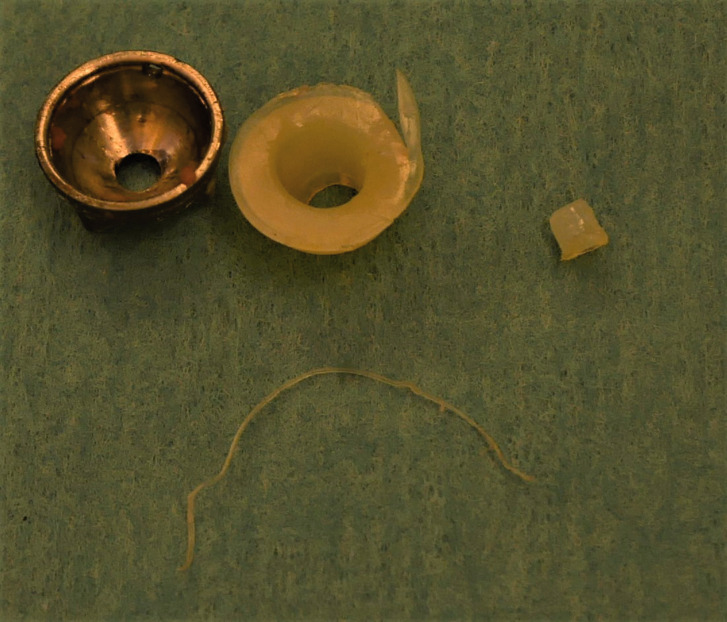
Patient 1: the excised Arpe® metal cup, the polyethylene liner with a strand that had broken off the outer perimeter and with a broken central peg.

A 57-year-old man experienced increasing pain on gripping at the base of his right dominant thumb 9 months after prosthetic implantation. There was slight clicking on adduction of the thumb, but no swelling, redness or increased temperature. Radiographs showed the head subsided in the cup (Figure 2(a) and (b)). Surgery revealed the liner loosened from the osseointegrated metal cup and the head perforating the PE liner (Figure 2(c)). The cup was replaced by the largest available size (10). After similar postoperative care he returned to work free of pain. Thirteen months after the revision his thumb became increasingly painful. CT scans showed the head articulating against the metal backing. At a second revision we found that the head again had perforated the loose liner. We removed the prosthesis and performed an arthrodesis.

**Figure 2.**
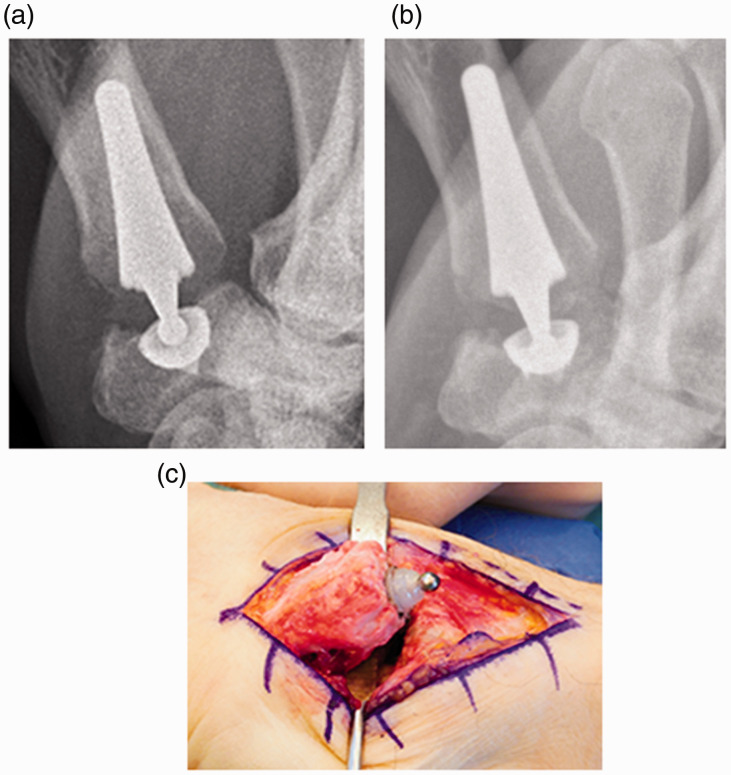
Patient 2: (a) 6 weeks after the original Arpe® implant; (b) 1 year postoperatively, the head has subsided into the cup; (c) the prosthetic head has penetrated through the polyethylene insert.

Dismantling of the Arpe® PE inlay seems rare. Even large series with long follow-up have not reported this complication (De Smet et al., 2020; Martin-Ferrero, 2014). Manufacturing small PE liners with metal backing is challenging because the liner has to be thin and the coupling between liner and metal can be a potential weakness (Figure 3). Thin PE liners are prone to stress concentration, cracking and breakdown (Shen et al., 2011). Fracture of the PE peg may also weaken the PE inlay and increase the effect of the stress concentration between the head and the rim of the central hole in the backing. This may lead to collapse of the PE and breakthrough of the head. Following the complications encountered in our two cases we have become cautious in recommending this prosthesis to patients with high load demands.

**Figure 3.**
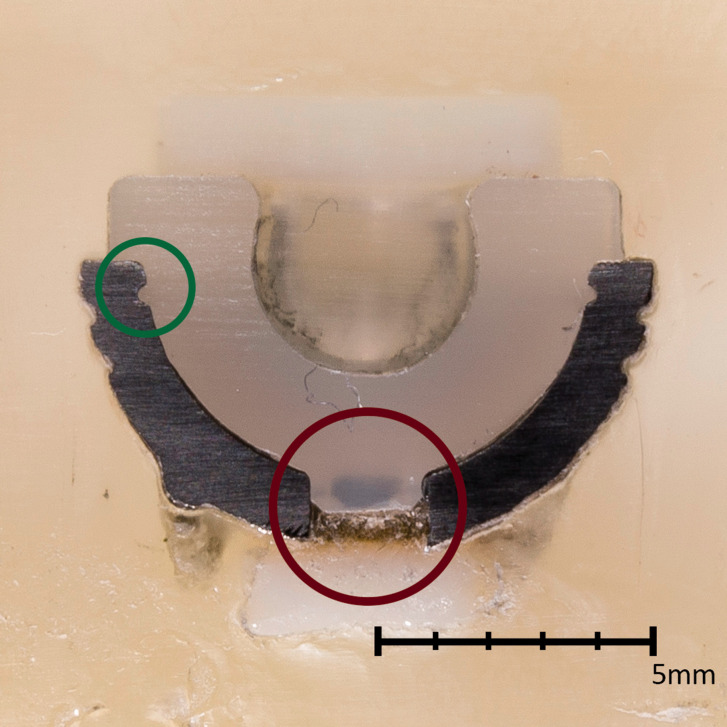
An unused and sectioned Arpe® cup embedded in resin; the stabilizing central polyethylene peg and the circumferential ring are circled in red and green, respectively.

## References

[bibr1-17531934211012203] De Smet A Vanhove W Benis S Verstraete M Hollevoet N . Ten-year outcomes of the Arpe prosthesis for the treatment of osteoarthritis of the trapeziometacarpal joint. Acta Orthop Belg. 2020, 86: 131–6. 32490784

[bibr2-17531934211012203] Martin-Ferrero M . Ten-year long-term results of total joint arthroplasties with Arpe® implant in the treatment of trapeziometacarpal osteoarthritis. J Hand Surg Eur. 2014, 39: 826–32. 10.1177/1753193413516244 24334554

[bibr3-17531934211012203] Shen FW Lu Z McKellop HA . Wear versus thickness and other features of 5-MRAD crosslinked UHMWPE acetabular liners. Clin Orthop Relat Res. 2011, 469: 395–404. 20848244 10.1007/s11999-010-1555-6 PMC3018202

